# Therapeutic potential of the MDM2 inhibitor Nutlin-3 in counteracting SARS-CoV-2 infection of the eye through p53 activation

**DOI:** 10.3389/fmed.2022.902713

**Published:** 2022-07-14

**Authors:** Giorgio Zauli, Sara AlHilali, Samar Al-Swailem, Paola Secchiero, Rebecca Voltan

**Affiliations:** ^1^Research Department, King Khaled Eye Specialistic Hospital, Riyadh, Saudi Arabia; ^2^Department of Translational Medicine and LTTA Centre, University of Ferrara, Ferrara, Italy; ^3^Department of Environmental and Prevention Sciences and LTTA Centre, University of Ferrara, Ferrara, Italy

**Keywords:** SARS-CoV-2, p53, Nutlin-3, MDM2 inhibitors, eye, cornea, tear film

## Abstract

Starting from the beginning of the severe acute respiratory syndrome Coronavirus-2 (SARS-CoV-2) global pandemic, most of the published data has concentrated on the respiratory signs and symptoms of Covid-19 infection, underestimating the presence and importance of ocular manifestations, such as conjunctivitis, usually reported in SARS-CoV-2 infected patients. With the present review we intend to resume the ocular involvement in SARS-CoV-2 infection and the recent discoveries about the different cell types and tissues of the eye that can be directly infected by SARS-CoV-2 and propagate the infection. Moreover, reviewing literature data about p53 expression in normal and diseased eye tissues, we hypothesize that the pleiotropic protein p53 present at high levels in cornea, conjunctiva and tear film might play a protective role against SARS-CoV-2 infection. Since p53 can be easily up-regulated by using small molecule non-genotoxic inhibitors of MDM2, we propose that topical use of Nutlin-3, the prototype member of MDM2 inhibitors, might protect the anterior surface of the eye from SARS-CoV-2 infection, reducing the spreading of the virus.

## Introduction

Under physiological conditions, p53 protein is maintained at low level in most normal tissues through a variety of mechanisms, mainly depending by the control mediated by its principal inhibitor murine double minute 2 (MDM2), also known as human double minute 2 (HDM2) in humans. MDM2 acts on p53 through a well-characterized negative feedback loop, and, when the negative regulatory role of MDM2 is lost, as often it happens in response to viral infections (1), the intracellular p53 levels increase, leading to biological effects which depend on the duration and the strength of p53 activation (2). p53 is a pleiotropic molecule deeply analyzed for its several functions and involvement in different pathways, ranging from cell cycle arrest and apoptosis induction to senescence modulation.

In the context of viral infection, it is emerging that human coronaviruses have developed several specific molecular ways to interfere with p53-mediated activities in infected cells. It has been shown that the viral papain-like proteases, PLPs, can act as MDM2 stabilizer *via* deubiquitination, leading to accelerated p53 proteasomal degradation (3, 4), and to a cascade of events which ensures the rapid growth of virus-infected cells due to the p53 loss. Analogously, the PLPs' mediated effect was demonstrated also on RCHY1, another E3 ubiquitin ligase involved in p53 ubiquitination, resulting in enhanced p53 degradation inside infected cells (5). Moreover, it was observed that knockout of *TP53* gene promoted viral replication, and that, on the opposite, the expression of p53 served as antiviral cellular molecule able to downregulate SARS-CoV replication (5). More recently, another group, screening the impact of SARS-CoV-2 proteins in several signaling pathways involved in viral infection, has demonstrated that the viral protease nsp5 can functionally repress p53 by interfering with its transcriptional activity, and has suggested p53 as an “intrinsic host restriction factor for the virus” (6). Similarly to Ma-Lauer, they have also observed that the overexpression of p53 significantly reduced virus production, sustaining the hypothesis of a fundamental role of p53 in managing the cellular antiviral defenses. These observations highlight the central role of p53 in controlling coronavirus replication inside infected cells. On these bases, we have recently suggested that Nutlin-3, or its oral version Idasanutlin, a potent and selective small-molecule inhibitor of MDM2, that promotes stabilization of p53, might be beneficial to treat the pulmonary infection induced by SARS-CoV-2 (7). Similarly, following the concept that restoring/inducing p53 may provide beneficial therapeutic results for infectious diseases, a group of the Georgetown University has proposed a *TP53* gene therapy approach for patients affected by COVID-19, hypothesizing the repurposing of the SGT-53 agent, an immunoliposome carrying a plasmid vector for TP53 expression, currently in phase II human trials for pancreatic cancer (8).

In this review, we aimed to focus the attention on the eye and investigate the potential role of the MDM2/p53 pathway, modulated by Nutlin-3, in counteracting/preventing SARS-CoV-2 ocular manifestations and diseases. To support this rationale, we reviewed the recent literature about the involvement of eye's tissues, cells and tears during SARS-CoV-2 infection, and resumed the data about MDM2 and p53 expression in ocular health and disease situations. Moreover, we analyzed the observations about the feasibility of p53 modulation through MDM2 inhibition in preclinical ocular models. Finally, we proposed a topical use of Nutlin-3 as a therapeutic approach to protect the anterior surface of the eye and to contrast SARS-CoV-2 infection and spreading. A reasoning on importance of cytokines modulation was also included.

## The anterior surface of the eye represents a gateway for SARS-CoV-2 infection

The main receptor for SARS-CoV-2, the angiotensin-converting enzyme 2, ACE2, is naturally present in the epithelial lining of the lower respiratory tract and digestive system (9). It serves as the main portal of entry for Covid-19 virus into the human body, leading to a variety of respiratory and gastrointestinal symptoms, but it has been detected in several cell types, with highest levels of expression in type II alveolar pneumocytes (10). Increasing experimental evidence has recently demonstrated that also ocular tissues express ACE2. Indeed, ACE2 receptor expression at the mRNA and protein level has been shown in multiple cell types involved in human vision, including cell types of the external surface of the eye (11–13). However, the proportion of cells in the ocular surface having greater-than-zero expression of ACE2 does not exceed 10% and the mean level of mRNA expression is approximately 0.6% (14). Although the expression level of ACE2 receptor seemed to be relatively low compared to the type II alveolar pneumocytes (15), epidemiological studies have reported ocular surface infection among the clinical manifestations of patients with COVID-19 (16); moreover, conjunctivitis has been reported as the initial presenting symptom in several cases (17). A systematic review and meta-analysis reported that approximately 10% of COVID-19 patients has ocular manifestations with at least one ocular symptom (such as dry eye, conjunctival injection/chemosis, tearing, itching and foreign body sensation), indicating also that attention to these manifestations is important in the detection of COVID-19 infection in the population (18).

Such evidence underscores the importance of the eye as a gateway for SARS-COV-2. Therefore, we will explore experimental and clinical evidence supporting the hypothesis that the ocular surface is a potential route for SARS-CoV-2 transmission.

## The tissues of the anterior surface of the eye express ACE2 and TMPRSS2 receptors

ACE2 has been detected in the epithelial cells of both skin and cornea, tissues that share the role of primary barrier against the external environment, having both an external stratified squamous epithelium, and that govern several patho-physiological responses including inflammation (19). In the anterior surface of the eye, as well as in the cornea, ACE2 has been found in the conjunctiva and limbus (20). Furthermore, it has been shown that cells of the same ocular surface tissues expressing ACE2 also express the co-receptor transmembrane serine protease 2 (TMPRSS2) (21), resulting susceptible to SARS-CoV-2 infection from a molecular point of view. Interestingly, from the clinical point of view, it has been shown that some SARS-CoV-2 patients presented conjunctivitis as first symptom, and sometimes as unique manifestation of the infection. Moreover, SARS-CoV-2 was detectable on the ocular surface (conjunctival swabs and tears) of both symptomatic and asymptomatic patients, and the isolated virus was infectious if used to infect cells *in vitro*, demonstrating that the eye can be infected and at the same time can support virus replication and spreading (21). Consistently with the hypothesis that the anterior surface of the eye might represent a potential route of infection, Casagrande detected SARS-CoV-2 genomic RNA in corneas of deceased patients affected by COVID-19 (22). Furthermore, Singh showed that SARS-CoV-2 can infect human conjunctival tissues, especially of inflamed conjunctiva (23), and observed that diseased conjunctival tissues, such as nevi, cyst, papilloma and polyps, overexpress ACE2 as compared to normal conjunctival tissues (24). Another study used a single-cell sequency approach to demonstrate that both ACE2 and TMPRSS2 genes were highly co-expressed in the goblet cells of the conjunctiva, along with genes involved in immunity process (25). Once established that the anterior surface of the eye represents a potentially important route of infection, it is noteworthy that the highest levels of SARS-CoV-2 viral entry-related proteins are present in the limbus (26, 27). ACE2 and TMPRSS2 were also described in post-mortem SARS-CoV-2 patient ocular surface tissues, in association with productive viral replication, most notably in limbal regions (26). Furthermore, the same authors described in whole-eye organoid model that conjunctival and limbal cells can be infected by the virus and that they support active viral replication. It is noteworthy that transcriptional analysis of *ex-vivo* infected ocular surface cells revealed robust induction of NF-κB in infected cells as well as diminished type I interferon signaling (26). These findings are particularly interesting since it has been clearly demonstrated the key role of p53 in activating type I interferon signaling and in down-regulating the NF-kB pro-inflammatory pathway (28).

In summary, corneal, limbal and conjunctival epithelia express ACE2 and TMPRSS2, can be infected by SARS-CoV-2 and support a productive infection, suggesting that the ocular surface is a potential route for the transmission of the virus, and it represents a possible risk of viral transmission, in particular for healthcare workers working at proximity to the eye and for recipient patients during the corneal transplantation procedures (29).

## Retinal cells can be infected by SARS-CoV-2

Although most of the experimental data available on this topic suggest the anterior surface of the eye as a possible site for SARS-CoV-2 entry and infection, other evidence suggests that also the retina can be infected. In this respect, Zhou demonstrated that ACE2 is expressed in several neuro-retinal cells and retinal vessels analyzed in post-mortem retinopathy specimens donated by non-diabetic and diabetic patients (30). In the same specimens, TMPRSS2 co-expression was detected in retinal neuronal cells, vascular and perivascular cells (30). Additional evidence of retinal involvement was provided by the detection of SARS-CoV-2 genomic RNA in retinal biopsies of deceased COVID-19 patients (31). These findings could explain the presence of retinal changes such as cotton wool spots and dot-blot hemorrhages reported in the literature (32). In line with the findings of Zhou, that observed a higher expression of ACE2 receptor in vessels of diabetic patients, Landecho showed that retinal microangiopathy might represent an *in vivo* biomarker of systemic vascular disease during SARS-CoV-2 infection (33). In fact, 6 out of 27 evaluated patients involved in the study showed cotton wool exudates, a marker of vascular disease severity in diabetes, associated with increased risk for acute vascular events. At present, it is not clear if these events can be caused directly by the virus acting in the endothelium of the vasculature or if they can be provoked by a disseminated intravascular hypercoagulable state (33). Moreover, if infection of the retina occurs and if it derives directly from the eye, from the upper respiratory tract or from another site are still open questions. Nonetheless, in the course of SARS-CoV-2 infection, it is very likely that ocular manifestations that affect the retina are modulated by an alteration of the blood-retinal barrier, BRB (34). This could occur at the level of the retinal endothelial cells of the inner barrier, iBRB, or at the level of retinal pigment epithelial, RPE, cells of the outer barrier, oBRB, or at both levels.

## Role of the tears/tear film and of the nasolacrimal duct morphology in the transmission of SARS-CoV-2 infection

During the first months of the pandemic, the viral RNA was not clinically evaluated in patients' tears, due to the fast spreading of the emergency, and rare eye symptoms were reported probably because underestimated compared to the more severe systemic symptoms (16, 35). Literature of later periods investigated the eye as possible route of infection, searching the presence of SARS-CoV-2 virus in tears, but results appeared highly variable (Table 1), leading authors to different conclusions about the relevance of SARS-CoV-2 RNA in tears. More recently, when data originated from studies where tears analysis was the main specific research target, and methods were standardized, the situation was clearer, indicating that tears/tear film collected by conjunctival swabs from moderate to severe COVID-19 affected patients contain viral RNA (Table 1). In the work of Arora and colleagues, SARS-CoV-2 RNA was detected in tears of 24% of patients with a confirmed COVID-19 infection, despite the absence of any ocular manifestation (38). In the study of Azzolini, viral RNA was detected in a larger part of the investigated cohort (57,1%), reporting low rate of ocular signs and indicating that in some patients the ocular surface can be positive when nasopharyngeal swab is negative (37). Interestingly, a small study reported preliminary data indicating tear film positivity in 72% of analyzed patients (36). This incredible high percentage derives probably by the characteristics of the investigated cohort that included patients with severe Covid-19 treated with full-mask or oxygen helmet. Indeed, the authors hypothesized that the dryness and eye irritation due to wearing the mask could facilitate the ocular surface infection by the high viral load present in the breath of pneumonia affected patients. Together, these evidence supports the hypothesis that viral shedding through the tear film is a potential route of SARS-CoV-2 transmission, even if tears are characterized by a specific content of constitutive antimicrobial molecules, such as lactoferrin and lysozyme. In particular, lactoferrin has been shown to possess antiviral activity against SARS-CoV-2 (45) and to be able to upregulate p53 through the activation of NF-kB (46), but its capacity of modulating viral spreading at physiological concentration needs to be evaluated.

**Table 1:** Overview of relevant literature data investigating SARS-CoV-2 in tears/tear film of infected patients.

**Aim of the study**	**Patients (n)**	**COVID-19 severity**	**Sampling method**	Sampling time^*^	**SARS-CoV-2 RNA in samples**	**Eye symptoms**	**Refs**
SARS-CoV-2 on ocular surfaces of sub-intensive patients with pneumonia	9	Severe	Conjunctival swab	<48h	72%	Bilateral conjunctivitis (78%)	Troisi et al. (36)
SARS-CoV-2 on ocular surfaces	91	Severe	Conjunctival swab	<48h	57.1%	Hyperemia (5.8%), secretions (5.8%), blepharitis (7.7%), other signs (13.5%)	Azzolini et al. (37)
SARS-CoV-2 on corneal disks	11	Severe	Conjunctival swab (post-mortem)	NA	45%	NA	Casagrande et al. (22)
SARS-CoV-2 in tears of patients with moderate and severe COVID-19	75	Moderate and severe	Conjunctival swab and/or Schirmer's test strip	<48 h	24%	None	Arora et al. (38)
SARS-CoV-2 on ocular surfaces (observational)	243	Mild, moderate and severe	Conjunctival swab	1–17 days	7%	NA	Gijs et al. (39)
Viral RNA in conjunctival secretion	49	Asymptomatic, mild and moderate (treated with antivirals)	Conjunctival swab	2–27 days	8.2%	None	Li et al. (40)
SARS-CoV-2 in tears	30	Mild and severe (treated with antivirals)	Conjunctival swab	1–16 days	3.3%	Conjunctivitis (3.3%)	Xia et al. (41)
Characteristics of ocular findings (retrospective)	38	Mild, moderate and severe	Conjunctival swab	NA	5.3%	Conjunctivitis (31.6%)	Wu et al. (42)
Viral RNA in conjunctival secretion	37	Mild and severe	NA	NA	2.7%	Conjunctival congestion (8.1%), eye inflammation (91.9%)	Liang et al. (43)
Viral shedding in tears	17	Mild	Schirmer's test strip	3–20 days	0%	None	Seah et al. (44)

* *Time after positive naso/oropharyngeal swab*.

The nasolacrimal ducts constitute the anatomical connection between the eye and the nose, permitting the direct communication between the ocular mucosal immune system and the associated lymphoid tissue of the nasal cavity, and contributing to the immunological interdependence between the ocular and the respiratory systems. Thus, the nasolacrimal system may function as a route for virus migration, either through drainage of tears through the duct to the respiratory tract or, vice-versa, from the upper respiratory tract through the nasolacrimal duct to the eye. Indeed, it has been hypothesized that after infecting the ocular surface, SARS-CoV-2 virus could enter the nasolacrimal ducts to infect the high respiratory tract (47). Moreover, other investigators indicated that the nasolacrimal system may provide an additional route of entry and infection of other tissues, including the epithelium of the lacrimal canaliculi, the nasolacrimal drainage system, the nasal passage, and the upper respiratory tract (48–50).

## MDM2 and p53 expression in healthy ocular tissues

Once established that the receptors for SARS-CoV-2 infection are present in different ocular tissues, it is of interest to elucidate whether these tissues express p53 and/or its major inhibitor MDM2. Although only few studies addressed the issue of the basal p53 expression in the eye, it is noteworthy that Tendler and Panshin recently demonstrated the presence of significant p53 protein levels both in the corneal epithelium as well as in the corneal tear film (51). Of note, they observed that while the concentration of p53 was low in the cytoplasm of most normal cell types, due to the potential harmful role of elevated p53 concentrations in inducing cell cycle arrest and/or apoptosis, abnormally high p53 content was detected by immunohistochemistry, Western blot analysis and electronic microscope examinations in corneal epithelial cells. These data confirmed previous results that reported strong p53 cytoplasmatic expression both in corneal as well as in conjunctival epithelium of mice (52). Differently from corneal epithelium, minute amount of p53 was found in retina, lens and iris. Consistently, MDM2 was identified in the retina, lens and iris while it was absent in the corneal epithelium (51). The abnormally high levels of p53 in the corneal epithelium and the absence of its negative regulator adds to corneal immune privilege (53, 54). In addition, it was demonstrated that a significant number of exosomes and other microvesicles containing p53 were present in the corneal mucin layer of the tear film (51). In a previous study, the same authors showed that, after ultraviolet irradiation, the cytoplasmic p53 protein in corneal epithelium cells became functionally active, following phosphorylation in Ser15, and moved from cytoplasm to nucleus (55).

Summarizing, it is possible to indicate that p53 is present at significant levels in the normal tissues of the anterior surface of the eye, with elevated concentration in the cornea, and it appears to be functional. Moreover, as mentioned above, high levels of p53 are expressed not only in the anterior surface of the eye but are present also in tear film, where p53 might play anti-neoplastic as well as anti-infection activities. Interestingly, a crosstalk has been demonstrated between the p53 pathway and lactoferrin (46), and both proteins are components of the tears. Together, they might represent a natural barrier against the development of tumors in the anterior surface of the eye (56), as well as against infections, and in particular against SARS-CoV-2 (57, 58).

## Modulation of p53 activity in pathological ocular tissues following MDM2-inhibition by Nutlin-3

In this section of the review we summarize literature data suggesting that the p53 activator Nutlin-3 can modulate the MDM2/p53 axis in the ocular tissues where co-expression of both ACE2 and TMPRSS2 has been demonstrated. As mentioned earlier, it is particularly noteworthy that p53 is abundantly expressed in normal eye tissues of cornea, limbus and conjunctiva, it's present in corneal tear film, and it may play a protective role against viral infections. In this respect, Nutlin-3 has been proposed as a potential pharmacological approach against pterygium, an anomalous non-tumoral proliferation of the conjunctiva invading limbus and cornea (59). Experiments based on primary cell lines, obtained after explant of pterygium tissues, have demonstrated that p53, highly expressed but not active and relegated to cytoplasm, translocated to the nucleus after treatment with Nutlin-3 and showed transcriptional activity and apoptotic effect. More recently, the therapeutic use of Nutlin-3 was showed in another disease of the conjunctiva, the conjunctival melanoma that typically express p53 in wild-type status. These authors demonstrated that Nutlin-3 was able to reactivate p53 and reduced viability in several models of conjunctival melanoma, both *in vitro* in cell lines and 3D spheroids, as well as *in vivo* in zebrafish xenografts (60). Considering the retina, an ocular formulation of Nutlin-3 was used in preclinical models of retinoblastoma, a pediatric disease characterized for expressing high levels of MDM2/HDMX (61). In this study, subconjunctival administration of Nutlin-3 improved intraocular penetration, respect to oral and iv administration, and exhibited specific p53-mediated antitumor effects. More recently, the use of Nutlin-3 was proposed to target and eliminate senescent RPE cells involved in the retinal degeneration phenomena occurring during the progressive and multifactorial disease known as age-related macular degeneration (AMD) (62). In this work, senescent RPE cells were shown to express high level of p53 and other expression markers of senescence like p21, p16, IL-1β, IL-6, and Mmp-3. Nutlin-3 treatment selectively killed more than 50% of senescent RPE cells, with no cytotoxicity on non-senescent cells, also decreasing the levels of released inflammatory cytokines, such as IL-6. This pharmacological approach was efficient both *in vitro* in Dox-induced senescent ARPE-19 cells, as well as *in vivo* in a mouse model of RPE senescence treated intravitreally with Nutlin-3. Interestingly, the authors observed also retinal regeneration and improving of retinal function in a model of aged mice, hypothesizing a possible therapeutic use of MDM2 inhibitors for this disease. Moreover, these data confirm the ability of Nutlin-3 to reduce the secretion of cytokines during senescence-associated secretory phenotype (SASP). Interestingly, in the context of retinal detachment secondary to proliferative vitreoretinopathy, Nutlin-3 was efficient in upregulate p53 expression of hyper proliferating RPE cells, limiting their growth and preventing retinal detachment (63). This observation leads to the important consideration that Nutlin-3 can modulate the proliferation of RPE cells when they are in an altered state, such as senescence or hyperproliferation, restoring a physiological situation.

So far, to our knowledge, beside conjunctiva, Nutlin-3 or other MDM2 inhibitors were not evaluated specifically to treat pathological cornea or limbus, the other ocular tissues expressing high levels of SARS-CoV-2 receptors. Differently, experimental uses of Nutlin-3 in diseases affecting other ocular tissues have been recently reviewed and further sustain the feasibility of its use as ocular treatment (64).

An important consideration regarding most of the works with a pharmacological use of Nutlin-3 for *in vivo* ocular treatment is that they were performed by intravitreal injection, an invasive method unfortunately needed for many eyes' disease therapies. A delivery method through eye drops, as was proposed by Brennan more than a decade ago, would be certainly easier for approaching preclinical studies, and more desirable and better accepted by future patients.

## Nutlin-3 might represent a new therapeutic tool to counteract SARS-CoV-2 infection of the eye

Following the here resumed evidence that external ocular tissues can be the portal of entry and site for spreading of SARS-CoV-2, and that p53 pathway can be activated in these tissues, it appears that a therapeutic intervention with Nutlin-3 can be feasible to counteract SARS-CoV-2 infection of the eye and merits to be pursued. In particular, Nutlin-3 subconjunctival formulations represent unique opportunities to efficiently deliver the drug topically, with a non-invasive method. Moreover, this approach could be further improved and targeted in the next future with the help of nano-formulations, such as lipid vesicles.

**Figure 1:**
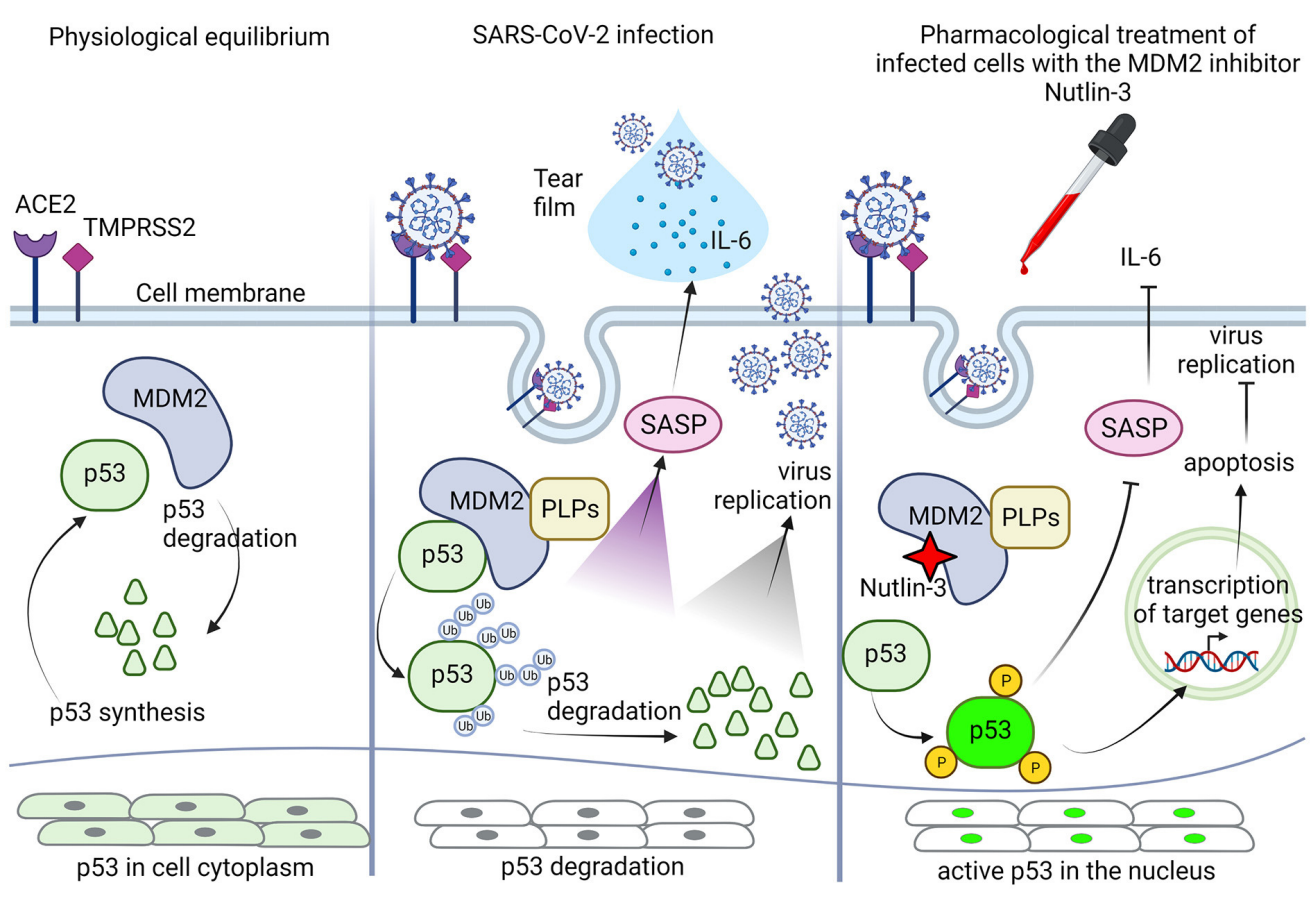
Schematic representation of the potential role of p53 in corneal epithelial cells in response to pharmacological treatment with Nutlin-3. At physiological equilibrium, p53 life in corneal epithelial cells is controlled by its inhibitor MDM2, p53 synthesis and degradation are in balance, and p53 protein is present in the cytoplasm at high level in an inactive status. During SARS-CoV-2 infection, the cellular physiological equilibrium is altered by the papain-like proteases (PLPs) of the virus, that stabilizing MDM2 promote p53 ubiquitination and degradation. The p53 inhibition allows virus to take control of intracellular events, promoting its own replication and eventually leading to activation of senescence-associated secretory phenotype (SASP), with extracellular secretion of IL-6 and presence of both IL-6 and mature virions in the corneal tear-film. Following pharmacological treatment with the MDM2 inhibitor Nutlin-3, possible by eye drops, Nutlin-3 can interact with MDM2 and can free p53 from its inhibition, blocking its degradation. PLPs have a different binding site on MDM2, so Nutlin-3 can work efficiently. Once free, p53 can be activated by phosphorylation and then migrate to the nucleus to repristinate the control over several pathways, including down regulation of SASP with inhibition of IL-6 secretion, and apoptosis induction through transactivation of specific target genes, to finally moderate/ inhibit virus replication and propagation. Created with BioRender.com.

A proposal about a possible combination of molecular mechanisms acting on corneal infected epithelium after pharmacological ophthalmic treatment with Nutlin-3 is presented in Figure 1. Since Nutlin-3 and coronavirus PLPs are competitors on MDM2, acting in opposite direction on the fate of p53, the first stabilizing the protein and the second promoting its degradation, the comprehension of the downstream mechanisms mediated by p53, other than the well-known apoptosis induction, and able to modulate infectivity and ocular symptoms are highly wished. Among these, considering that the “cytokine storm” is an important clinical manifestation during the severe SARS-CoV-2 infections (35), one of the potential beneficial anti-viral mechanisms of promoting p53 activation could be to reduce the senescence-associated secretory phenotype and the interleukin-6 secretion. Indeed, SASP is negatively controlled by p53 (65), and it was demonstrated that SARS-Cov-2 infection can promote both IL-6 secretion in tears (39, 66), and inflammatory response in human corneal epithelial cells (67). Nutlin-3 treatment could then drive SASP inhibition and IL-6 secretion, through the inhibition of MDM2 and upregulation of p53. Moreover, since ACE2 expression is upregulated in inflamed eye's tissues (24, 68), reducing IL-6 could be a strategy to reduce local inflammation and to restore baseline receptor levels.

Other mechanisms could involve the transcriptional modulation of ACE2 and TMPRSS2, and the activation of the innate immune response. In the last context, it is known that p53 participates to the antiviral innate immune responses by inducing apoptosis of infected cells and by mediating type I interferon, IFN-I, synthesis/signaling (3, 28). On the other hand, it was demonstrated that SARS-CoV-2 can manipulate the host innate immune response blocking or inducing IFN-I pathway to its advantage, and it is sensitive to IFN treatment (63, 69). How p53, enhanced by Nutlin-3, could win the battle is a new challenge. Interestingly, topical administration of IFN is largely used in ophthalmic practice for treatment of several ocular surface disorders, and its therapeutic and side effects are well known (70, 71). In the spectrum of the responses induced thanks to the activation of p53, topical use of Nutlin-3 could also induce type I interferon signaling (3, 28), having the limitation compared to recombinant interferon of a less direct and focused action, but the advantage of being broader, with the activation of the innate immune response along with other effects discussed above. Obviously, this specific ability remains to be preclinically characterized at the ocular level. In parallel, it should be interesting to evaluate the efficacy of IFN-I local therapy in the eye that, to our knowledge, has not being investigated against SARS-CoV-2 so far.

We believe that all these considerations pose the basis for a pharmacological approach against ocular SARS-CoV-2 infection mediated by the control of p53 expression with Nutlin-3 as preferred candidate.

## Author contributions

Conceptualization: GZ. Writing original draft preparation and writing review and editing: GZ, SA, SA-S, RV, and PS. All authors have read and agreed to the published version of the manuscript.

## Funding

Funds for open access publication fees were received by RV as local funding from University of Ferrara (FAR and FIR programs).

## Conflict of interest

The authors declare that the research was conducted in the absence of any commercial or financial relationships that could be construed as a potential conflict of interest.

## Publisher's note

All claims expressed in this article are solely those of the authors and do not necessarily represent those of their affiliated organizations, or those of the publisher, the editors and the reviewers. Any product that may be evaluated in this article, or claim that may be made by its manufacturer, is not guaranteed or endorsed by the publisher.
